# Effective SARS-CoV-2 replication of monolayers of intestinal epithelial cells differentiated from human induced pluripotent stem cells

**DOI:** 10.1038/s41598-023-38548-1

**Published:** 2023-07-18

**Authors:** Shohei Minami, Naomi Matsumoto, Hiroko Omori, Yutaka Nakamura, Shigeyuki Tamiya, Ryotaro Nouda, Jeffery A. Nurdin, Moeko Yamasaki, Tomohiro Kotaki, Yuta Kanai, Toru Okamoto, Taro Tachibana, Hiroshi Ushijima, Takeshi Kobayashi, Shintaro Sato

**Affiliations:** 1 Department of Virology, Research Institute for Microbial Diseases, Osaka University, Osaka, 565-0871 Japan; 2 Core Instrumentation Facility, Research Institute for Microbial Diseases, Osaka University, Osaka, 565-0871 Japan; 3 Department of Microbiology and Immunology, School of Pharmaceutical Sciences, Wakayama Medical University, Wakayama, 640-8156 Japan; 4 Institute for Advanced Co-creation Studies, Research Institute for Microbial Diseases, Osaka University, Osaka, 565-0871, Japan; 5 Center for Infectious Disease Education and Research, Osaka University, Osaka, 565-0871 Japan; 6 Cell Engineering Corporation, Osaka, 532-0011 Japan; 7 Department of Bioengineering, Graduate School of Engineering, Osaka Metropolitan University, Osaka, 558-8585 Japan; 8 Division of Microbiology, Department of Pathology and Microbiology, Nihon University School of Medicine, Tokyo, 173-8610 Japan

**Keywords:** Infection, SARS-CoV-2, Experimental models of disease

## Abstract

Severe acute respiratory syndrome coronavirus 2 (SARS-CoV-2) causes severe acute respiratory symptoms in humans. Controlling the coronavirus disease pandemic is a worldwide priority. The number of SARS-CoV-2 studies has dramatically increased, and the requirement for analytical tools is higher than ever. Here, we propose monolayered-intestinal epithelial cells (IECs) derived from human induced pluripotent stem cells (iPSCs) instead of three-dimensional cultured intestinal organoids as a suitable tool to study SARS-CoV-2 infection. Differentiated IEC monolayers express high levels of angiotensin-converting enzyme 2 and transmembrane protease serine 2 (TMPRSS2), host factors essential for SARS-CoV-2 infection. SARS-CoV-2 efficiently grows in IEC monolayers. Using this propagation system, we confirm that TMPRSS2 inhibition blocked SARS-CoV-2 infection in IECs. Hence, our iPSC-derived IEC monolayers are suitable for SARS-CoV-2 research under physiologically relevant conditions.

## Introduction

Severe acute respiratory syndrome coronavirus 2 (SARS-CoV-2) belongs to the genus *Betacoronavirus* of the family *Coronaviridae*^1^. SARS-CoV-2 was first isolated from a patient with severe respiratory disease in 2019. Since then, SARS-CoV-2 has spread worldwide in a few months. To date, the coronavirus disease (COVID-19) pandemic is still not under control^2^.

The SARS-CoV-2 particle includes the spike glycoprotein (SP), the receptor binding protein. The SP forms trimers in the virus particle and is divided into the S1 and S2 regions. S1 contains the receptor-binding domain. The receptor of SARS-CoV-2, angiotensin-converting enzyme 2 (ACE2), has been identified to bind to the receptor-binding domain^3^. During entry and release of SARS-CoV-2, the SP is cleaved by several host proteases, such as FURIN, cathepsin-L (CTSL), and transmembrane protease serine 2 (TMPRSS2)^4,5^. In humans, ACE2 is distributed not only in the lung but also in the heart, kidney, intestine, and bladder, leading to various symptoms in individuals infected with SARS-CoV-2^6^. ACE2 is highly expressed in the intestine, suggesting that SARS-CoV-2 easily replicates in intestinal cells^7,8^.

Since the establishment of induced pluripotent stem cells (iPSCs)^9^, the number of studies using human iPSCs has drastically increased. The cells have been used to analyze the tropism of SARS-CoV-2. A recent study showed that SARS-CoV-2 infects monolayers of differentiated human lung cells derived from iPSCs and induces an inflammatory response^10^. Three-dimensional (3D)-cultured human lung, cerebral, and intestinal organoids derived from iPSCs could also be infected with SARS-CoV-2^11,12^. Other previous studies demonstrated SARS-CoV-2 infection in 3D-cultured human small intestinal organoids derived from primary gut epithelial cells and 2D-cultured human alveolar type II-like cells derived from primary epithelial lung cells, which express ACE2^13,14^. However, the biological features of alveolar cells were not suitable to analyze SARS-CoV-2 infection^13,15,16^. Recently, the bronchial organoid-derived air–liquid interface model (BO-ALI) improved the growth of SARS-CoV-2 in bronchial organoids^17^; however, both lung organoids and BO-ALI require complicated handling technics and a high maintenance cost. These findings showed that 3D-cultured intestinal organoids are useful for studying SARS-CoV-2. However, since 3D-cultured epithelial cells maintain polarity, such that the luminal side is usually on the inside, they require a digestion step prior to virus infection, after which the cells must form 3D-cultured organoids again in the Matrigel. Therefore, infecting 3D-cultured organoids is complicated. In contrast, 2D-cultured cells are easy to handle compared with 3D-cultured cells. Recently, it has been reported that the expression levels of intestinal markers in 2D-cultured monolayers are conserved compared with those in 3D-cultured organoids^18,19^.

Many high-throughput drug screenings against SARS-CoV-2 have been published^20–24^. However, most of these reports used the African green monkey cell line, Vero cells, or 3D-cultured human organoids. Although the efficacies of compounds could be demonstrated in Vero cells, additional in vivo experiments were needed. The most important animal model of SARS-CoV-2 infection is the Syrian hamster (*Mesocricetus auratus*). SARS-CoV-2 replicates well in these hamsters^25^, but effective in vitro SARS-CoV-2 replication in physiologically normal cells is also necessary. Such in vitro propagation systems are particularly essential for high-throughput drug screening. However, using 3D-cultured human organoids or 2D-cultured human alveolar type II-like cells did not reproduce severe COVID-19 due to the inefficient growth of SARS-CoV-2 in these systems^10–14^.

In this study, considering the simplicity of operation and the importance of using normal human cells, we present an efficient in vitro SARS-CoV-2 propagation system using 2D-cultured human iPSC-derived intestinal epithelial cells (IECs) (Supplementary Table 1). We believe that this method will enable high-throughput drug screening under physiological conditions in the future.

## Results

### Characterization of human iPSC-derived IECs

**Figure 1:**
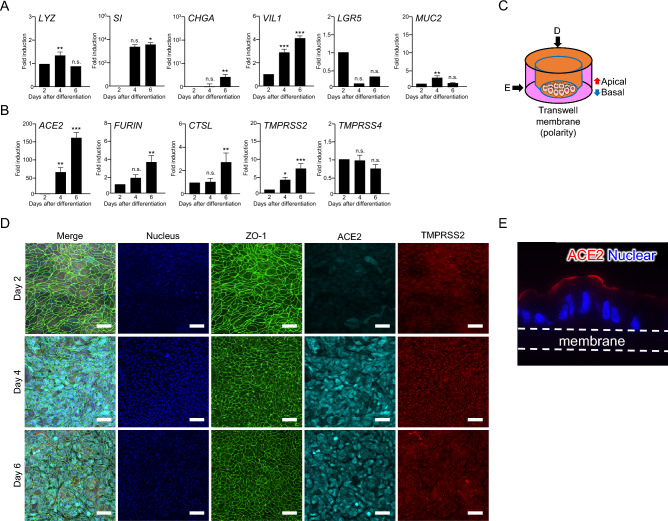
The characteristics of the IEC#17. The IEC#17 monolayers were cultured in a differentiation medium from day 2. **(A,** **B)** IEC#17 cells were collected at the indicated time points. Relative mRNA expression of the indicated genes in IEC#17 during the course of differentiation (days 2, 4, and 6) were determined by qRT-PCR and normalized against the expression of *GAPDH*. Each result was normalized by the expression level at two-day after differentiation. Each value is representative of at least three independent experiments and is shown as the mean ± SD from three wells of cells of each culture group. The significant differences were determined using one-way ANOVA. **(A)** The transcription of the major intestinal markers was measured using qRT-PCR. We included *LYZ*, *SI*, *CHGA*, *VIL1*, *LGR5* and *MUC2* which is the marker of Paneth cell, enterocyte, enteroendocrine cell, IEC, stem cell and goblet cell, respectively. **(B)** The transcription of the major host factors important for SARS-CoV-2 infection was measured using qRT-PCR. We included *ACE2*, *FURIN*, *CTSL*, *TMPRSS2* and *TMPRSS4*. **(C)** Schematic diagrams of the immunostaining analysis. The black arrow indicates the direction from which the photograph was taken. **(D)** The protein expressions of ZO-1, ACE2, and TMPRSS2 in IEC#17 monolayers were visualized by immunofluorescence. IEC#17 monolayers were fixed and stained at the indicated time points. The scale bar indicates 50 µm. **(E)** The protein expressions of ACE2 in IEC#17 monolayers with polarity were visualized by immunofluorescence. The photo was taken from vertical angle at six-day after differentiation. The white broken bar indicates Transwell membranes. *0.01 < *P* < 0.05; **0.005 < *P* < 0.01; ***P < 0.005.

3D-cultured IECs, IEC#17, were trypsinized and then seeded in a 96-well plate. First, we checked the transcription of intestinal markers in IEC#17 using quantitative reverse transcription-polymerase chain reaction (qRT-PCR). IEC#17 generally expressed *sucrase isomaltase* (*SI*) and *villin1* (*VIL1*), both of which are marker genes for enterocytes (Fig. 1A), indicating that IEC#17 well differentiated into IECs as we reported previously^18,19^.

Next, we checked the expression levels of the host factor genes involved in SARS-CoV-2 infection, such as *ACE2*, *FURIN*, *CTSL*, *TMPRSS2*, and *TMPRSS4*, in IEC#17 monolayers, using qRT-PCR. During IEC differentiation, the mRNA expression of each gene gradually increased (Fig. 1B); particularly, *ACE2* transcription drastically increased. Moreover, the increase in *TMPRSS2* transcription was remarkable. *FURIN* and *CTSL* transcriptions were also increased, but *TMPRSS4* transcription did not increase. The protein expressions of ACE2 and TMPRSS2 in IEC monolayers on days 2, 4, and 6 post-differentiation were confirmed by immunofluorescence staining in the horizontal or vertical plane (Fig. 1C−E). Although ACE2 protein expression was consistent with the mRNA expression, TMPRSS2 protein expression seemed to be lower than the mRNA expression (Fig. 1B,D). ACE2 was mainly located on the cell surface at the luminal side in polarized IEC#17 monolayers (Fig. 1E). Taken together, these data suggested that fully differentiated IEC#17 monolayers (6 days post-differentiation) expressed SARS-CoV-2-related genes, indicating their potential for effective SARS-CoV-2 infection.

### SARS-CoV-2 growth in human iPSC-derived IECs

**Figure 2:**
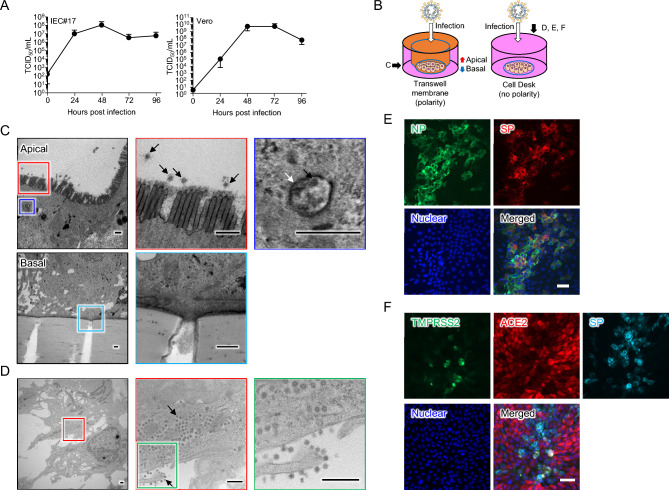
The potential of IEC#17 for the efficient SARS-CoV-2 infection. **(A)** IEC#17 and Vero cells were seeded in a plate and infected with SARS-CoV-2 at an MOI of 0.1. The supernatants were collected at indicated time points. SARS-CoV-2 growth was measured by determining the TCID_50_ on VeroE6/TMPRSS2 cells. Each value is representative of at least three independent experiments and is shown as the mean ± SD from three wells of supernatants of each culture group. The significant differences were determined using two-way ANOVA. **(B**–**D)** The transmission electron microscopic analysis of IEC#17 monolayers infected with SARS-CoV-2. IEC#17 cells were seeded on Transwell membranes **(C)** or Cell Desks **(D)**. Each colored squares indicate each magnified panels. **(B)** Schematic diagrams of the transmission electron microscopic analysis. The black arrow indicates the direction from which the photograph was taken. **(C)** The IEC#17 were cultured to harbor the polarity and infected with SARS-CoV-2 at an MOI of 0.1 from apical side. At 24 hpi, infected cells were fixed and analyzed. The photos were taken from vertical angle. The black and white arrows indicate SARS-CoV-2 particle and viral replication organelles (ROs), respectively. The scale bars indicate 0.5 µm. **(D)** The IEC#17 were cultured by the normal method (no polarity) and infected with SARS-CoV-2 at an MOI of 0.1. At 24 hpi, infected cells were fixed and analyzed. The photos were taken from horizontal angles. The black arrows indicate SARS-CoV-2 particle. The scale bars indicate 0.5 µm. **(E**, **F)** Immunofluorescence analysis of **(E)** SARS-CoV-2 NP and SP, or **(F)** ACE2, TMPRSS2, and SARS-CoV-2 SP in IEC#17 monolayers infected with SARS-CoV-2. The photo was taken from horizontal angles at 24 hpi. The scale bars indicate 50 µm.

Although it has been reported that enterocytes in human small intestinal organoids are susceptible to SARS-CoV-2 infection^12,13^, the susceptibility of 2D-cultured IEC monolayers to SARS-CoV-2 infection has not been evaluated. To investigate the infection of SARS-CoV-2 in IEC#17 monolayers, IEC#17 and Vero cells were seeded in 96-well and 24-well plates, respectively. The cells were infected with SARS-CoV-2 at a multiplicity of infection (MOI) of 0.1. At each time point, supernatants were collected, and the viral titers were measured by determining the median tissue culture infectious dose (TCID_50_). SARS-CoV-2 grew well in both cell types, and the viral titers peaked at 48 h post-infection (hpi) (Fig. 2A). Although the peak viral growth rate was higher in Vero cells (ΔTCID_50_/mL: 10^9^ vs. 10^6^), IEC#17 showed comparable levels of viral growth to Vero cells at 24 hpi (ΔTCID_50_/mL: ≈10^5^). In addition, the replication rate of the virus in IEC#17 was remarkably higher than that previously reported in human cell lines or organoids^12–14,25^.

SARS-CoV-2 particles were visualized using transmission electron microscopy (TEM). IEC#17 cells were seeded onto Matrigel-coated Transwell membranes or plastic coverslips (Cell Desk) and infected with SARS-CoV-2 at an MOI of 0.1. The infected cells in Transwells and Cell Desks were observed vertically and horizontally, respectively (Fig. 2B). SARS-CoV-2 particles were visible in IEC#17 monolayers from both angles (Fig. 2C,D). Budding virus particles were mainly observed at the apical side of the IEC#17 cells (Fig. 2C, red square). Viral replication organelles (ROs) were also observed (Fig. 2C, blue square), indicating an active viral cycle. Although some secretions were observed to be released from the pore in the Transwell membrane, budding virus particles were not observed at the basal side of the IEC#17 cells within our field of view (Fig. 2C, cyan square). Horizontally, several SARS-CoV-2 particles that replicated inside the cells were clearly observed per cell (Fig. 2D). Budding virus particles were also observed. Although clear visible ROs composed of a membrane were not observed, we found assembled viruses (Fig. 2D). These observations indicated that SARS-CoV-2 replication and budding were active in IEC#17. Further, immunostaining revealed the SARS-CoV-2 nucleoprotein (NP) and SP in post-infected IEC#17 (Fig. 2E). Interestingly, SP-positive IECs appeared to have downregulated expression of ACE2 (Fig. 2F). These data supported that SARS-CoV-2 could infect and replicate in IEC#17 monolayers. Therefore, we propose that IEC#17 is suitable for SARS-CoV-2 research.

### Comparison of the propagation efficiency of SARS-CoV-2 among IEC#17 cells, other iPSC-derived IECs, and tissue-derived primary IECs

**Figure 3:**
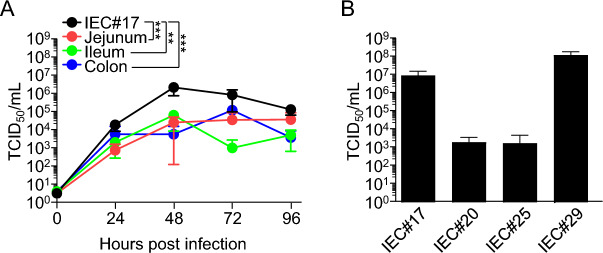
Comparison of SARS-CoV-2 growth in IEC#17 and other IECs. **(A)** The growth of SARS-CoV-2 was compared in intestinal epithelial cells differentiated from induced pluripotent stem cells (iPSCs) (IEC#17; black) and primary IECs derived from jejunum (red), ileum (green) and colon (blue). Cells were infected at an MOI of 0.1. The supernatants were collected at the indicated time points. SARS-CoV-2 growth was measured by determining the TCID_50_ on VeroE6/TMPRSS2 cells. Each value is representative of at least three independent experiments and is shown as the mean ± SD from three wells of supernatants of each culture group. The significant differences were determined using two-way ANOVA. **(B)** SARS-CoV-2 titers in IECs differentiated from various iPSCs. The IECs were infected with SARS-CoV-2 at an MOI of 0.1. The supernatants were collected at 48 hpi. SARS-CoV-2 growth was measured by determining the TCID_50_ on VeroE6/TMPRSS2 cells. Each value is representative of at least three independent experiments and is shown as the mean ± SD from three wells of supernatants of each culture group. *0.01 < *P* < 0.05; **0.005 < *P* < 0.01; ***P < 0.005.

It has been reported that SARS-CoV-2 can replicate in tissue-derived 3D-cultured IECs^13^. We directly compared the growth of SARS-CoV-2 in primary IECs derived from jejunum, ileum, and colon tissues with that in IEC#17 cells, which were in a monolayered state. The viral titers in all primary-derived IECs were significantly lower than those in IEC#17 (Fig. 3A). This suggests that IECs derived from human iPSCs are more susceptible to SARS-CoV-2 infection than tissue-derived IECs.

To confirm the effective replication of SARS-CoV-2 caused by the culture system of iPSC-derived IEC monolayers, we next investigated the possibility of in vitro propagation of SARS-CoV-2 in other IECs differentiated from other human iPSC lines. We established IEC#20, IEC#25, and IEC#29 from the human iPSC lines 1231A3, 1383D6, and TkPP7, respectively. Since the viral titers reached the peak at 48 hpi in IEC#17 (Fig. 2A), we comparatively analyzed the viral titers at 48 hpi in these IECs. Although the viral titers in IEC#29 were as high as those in IEC#17, they were lower in IEC#20 and IEC#25 cells (Fig. 3B). To determine the characteristics of these IECs, qRT-PCR and immunostaining were conducted. The results showed that there were no marked differences in IEC #20 and IEC #25 compared with those in IEC #17, except for a slight increase in *CHGA* expression (Supplementary Fig. 1). IEC#29 also showed gene expression changes similar to those of IEC#17, in addition to exhibiting a slight increase in *MUC2* expression. The protein expression of ACE2 and TMPRSS2 was increased at 6 days post-differentiation, similar to the observation in IEC#17; however, the expression of ACE2 in IEC#20 was lower than that in the other IECs (Supplementary Fig. 2). These results suggest that not only IEC#17 but also IEC#20, #25, and #29 have the characteristics of intestinal epithelial cells, and that SARS-CoV-2 can grow in vitro in several different iPSC-derived IECs.

### Influence of the inhibition of TMPRSS2 in IEC#17

**Figure 4:**
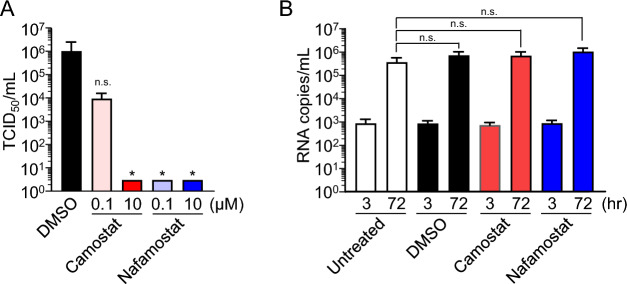
Testing the effect of TMPRSS2 inhibitors on SARS-CoV-2 replication in IEC#17. **(A)** IEC#17 monolayers were treated with either DMSO or 0.1 µM or 10 µM camostat and nafamostat for 1 h. After incubation, the pretreated cells were infected with SARS-CoV-2 at an MOI of 0.1. The supernatants of infected IEC#17 were collected at 48 hpi. SARS-CoV-2 titers were measured by determining the TCID_50_. Each value is representative of at least three independent experiments and is shown as the mean ± SD from three wells of supernatants of each culture group. The significant differences were determined using one-way ANOVA. The significant differences were determined using one-way ANOVA. **(B)** IEC#17 monolayers were untreated or treated with DMSO, 10 µM camostat and nafamostat for 1 h. After incubation, the pretreated cells were infected with 2 × 10^6^ genome equivalents of norovirus GII.4. The supernatants of infected IEC#17 cells were collected at 3 and 72 hpi. The viral RNA copies of norovirus GII.4 in IEC#17 were measured by qRT-PCR. Each value is representative of at least three independent experiments and is shown as the mean ± SD from between four and six wells of supernatants of each culture group. The significant differences were determined using one-way ANOVA. *0.01 < *P* < 0.05.

The most important advantage of using IEC#17 monolayers for SARS-CoV-2 research is their ability to replicate the in vivo condition and perform quantitative cellular assays. To investigate the interferon (IFN) response, mRNA transcripts in IEC#17 infected with SARS-CoV-2 were quantified by qRT-PCR. As the IFN response against SARS-CoV-2 in IEC#17 was normal (Supplementary Fig. 3), IEC#17 could be expected to be available for several analyses. To evaluate whether IEC#17 monolayers are suitable for drug screening, cells were pretreated with the TMPRSS2 inhibitors camostat and nafamostat before infection with SARS-CoV-2. These inhibitors prevent the cleavage of the SP, which is required for the membrane fusion of SARS-CoV-2 with the host cell, thereby inhibiting viral replication. As expected, both inhibitors completely inhibited SARS-CoV-2 infection at a concentration of 10 µM (Fig. 4A). Nafamostat can also inhibit replication at a concentration of 0.1 µM. To confirm the conservation of cell properties, the inhibitory assay was conducted using human norovirus (NoV) as a negative control. Propagation of human NoV GII.4, which infects IECs in a TMPRSS2-independent manner, was not affected by the two inhibitors (Fig. 4B). Altogether, these findings suggest that monolayered IEC#17 cells are a suitable alternative to alveolar epithelial type II cells and that IECs more closely resemble native cells than Vero cells.

## Discussion

Recently, the number of studies investigating SARS-CoV-2 in vitro and in vivo has drastically increased. However, most in vitro studies use Vero cells or human cells with forced expression of ACE2 (e.g., HEK293T/ACE2) as SARS-CoV-2 grows efficiently in these cells. SARS-CoV-2 also grows in several human cell lines, such as Calu-3 and Caco-2. These cell lines highly express ACE2, the receptor of SARS-CoV-2. Pseudotyped vesicular stomatitis virus containing the SARS-CoV-2 SP grows to comparable titers in these cell lines^26^. 3D-cultured IECs and lung organoids and monolayered alveolar type II-like cells are also susceptible to SARS-CoV-2 infection^10–14^. However, SARS-CoV-2 grew much less effectively in these human primary cells than in Vero cells. Our established IEC#17 monolayers enabled SARS-CoV-2 to grow effectively; however, the peak viral titer in IEC#17 was still lower than that in Vero cells. Although the well-differentiated IECs are unable to grow further, some of the non-infected or low-infected Vero cells grow and can support virus replication. Thus, the viral titer of SARS-CoV-2 in Vero cells was higher than that in IEC#17. In addition, it should be noted that Vero cells are type I IFN-deficient^27^. The circular polymerase extension reaction, a great tool for producing recombinant SARS-CoV-2, was established using HEK293-3P6C33 cells, in which IFN alpha and beta receptor subunit 1 (IFNAR1) has been knocked out^28^. These findings indicate that SARS-CoV-2 grows better in cells with an impaired IFN signaling is required to grow SARS-CoV-2 to high titers. Interestingly and importantly, IEC#17 monolayers induced type I IFNs, especially *IFN alpha* (*IFNA*), in response to SARS-CoV-2, indicating that IEC#17 has an intact IFN response. These observations could explain why SARS-CoV-2 grew better in Vero cells than in IEC#17. Therefore, knockdown or knockout of the *IFNAR1* gene might further enhance viral replication in IEC#17. Furthermore, IEC#17 appears to be a suitable human cell model to analyze type I IFN responses induced by SARS-CoV-2.

Although previous studies have reported SARS-CoV-2 infection in 3D-cultured IEC organoids^12,13^, the monolayers from iPSC-derived IECs showed a more efficient SARS-CoV-2 growth. The difference between previous reports and our findings might be because we used iPSC-derived IEC monolayers since pluripotent stem cell-derived IECs have been reported to be similar to fetal, but not adult, IECs^29^. It was suggested that differentiated fetal IECs are more susceptible to SARS-CoV-2 infection than differentiated adult tissue-derived IECs. Notably, fetal-derived and/or pluripotent stem cell-derived IECs are more susceptible to SARS-CoV-2 infection, but in general, COVID-19 is more likely to cause severe symptoms in the elderly^30–32^.

3D-cultured organoids serve as an excellent model to reflect in vivo conditions. However, as with bronchial epithelial cells^33^, SARS-CoV-2 is expected to easily invade and bud from the apical side of IECs. Therefore, 3D-cultured organoids will be slightly difficult to analyze for apical infection, and 2D-cultured monolayer cells seem to be more suitable than organoids. We previously reported that the expression levels of several intestinal markers and functional genes of IECs do not differ between 2D- and 3D-cultured conditions^18^. Although the reasons why the growth of SARS-CoV-2 was better in the monolayer IECs than 3D-cultured ones are still unclear, one possibility is that the luminal side of 3D-cultured IECs is enclosed, and replicating viruses and cellular metabolites accumulate in a limited space, leading to a rapid increase in their concentration. SARS-CoV-2 is an enveloped virus, which is sensitive to unfavorable environmental conditions, such as unsuitable pH^1^. Therefore, the luminal side of 3D-cultured IECs might die faster after viral infection than that of 2D-cultured IECs. Thus, the 2D-cultured monolayer IECs will contribute to a more easy and effective analysis of the virus.

SARS-CoV-2 growth in tissue-derived IECs was lower even under 2D culture conditions than that in IEC#17 monolayer. Regarding the comparison with primary cultured cells, we have recently reported that the characteristics of IECs in monolayer culture are indistinguishable between those derived from iPSCs (TkDN4-M) and those derived from small intestinal tissue^19^. In addition, SARS-CoV-2 growth varied in IEC monolayers differentiated from different iPSCs. Although the expression of SARS-CoV-2 infection-related genes was analyzed, critical differences were not observed among various IECs. Histo-blood group antigens are highly expressed in the intestine, and some enteric viruses (e.g., NoV and rotavirus) use these glycans for infection^34,35^. The H antigen expression in IECs requires fucosyltransferase 2 (FUT2) activity. IEC#17 and IEC#29 were derived from human iPSCs whose FUT2 activity is intact. In contrast, the *FUT2* gene in IEC#20 and #25 is mutated, rendering it inactive (Sato et al., unpublished data). Thus, the fucosylation on the apical side of IECs by FUT2 might influence the susceptibility to SARS-CoV-2. In addition, iPSCs may differ in their properties as pluripotent stem cells depending on the origin of the original somatic cells and the method of reprogramming, which may affect the properties of the differentiated IECs. However, further studies are required to elucidate the differences in responsiveness of different IECs to SARS-CoV-2 infection and propagation.

In conclusion, differentiated human iPSC-derived IECs might reflect the in vivo condition better than Vero cells, which are type I IFN-deficient monkey cells. SARS-CoV-2 grew better in 2D-cultured IEC#17 cells than in 3D-cultured IECs and 2D-cultured primary IECs. In vitro assays using IEC#17 monolayers are the easiest way to characterize severe COVID-19. Furthermore, IEC#17 monolayers are useful for screening drugs and neutralization antibodies and investigating the type I IFN response during SARS-CoV-2 infection.

## Materials and methods

### Cells

The African green monkey kidney cell line, Vero cells (ATCC®CCL-81™), were maintained in Dulbecco’s modified Eagle’s medium (DMEM; Nacalai Tesque, Kyoto, Japan) containing 5% fetal bovine serum (FBS; Thermo Fisher Scientific, MA, USA). Vero cells expressing TMPRSS2, VeroE6/TMPRSS2 cells (VeroE6/TMPRSS2, JCRB number; JCRB1819), were maintained in DMEM containing 5% FBS and 1 mg/mL G418 disulfate aqueous solution (Nacalai Tesque) at 37 ℃. IEC#17 cells were differentiated from a human iPSC line, TkDN4-M, as described previously^33^. IEC#20, IEC#25, and IEC#29 were also differentiated from the human iPSC lines, 1231A3, 1383D6^36^, and TkPP7^37^, respectively. To prepare monolayers, IECs were cultured in a multiwell plate at a density of 6.3 × 10^5^/cm^2^ in a culture medium for 2 days. For differentiation, cells were cultured in a differentiation medium for 4 days (for details, refer to Ref.^38^). After 6 days, an average of 1 × 10^5^ differentiated IECs were seeded in 96-well plates.

### Viruses

The ancestral SARS-CoV-2/Hu/DP/Kng/19-020 strain (GenBank accession no. LC528232; lineage B) was propagated in VeroE6/TMPRSS2 cells. The supernatant of infected cells at an MOI of 0.1 was collected at 48 hpi and centrifuged at 1600×*g* at 4 ℃ for 5 min to remove cell debris. The supernatant was stocked at − 80℃ until used. The viral titer was determined using the TCID_50_ method, as described below. To inoculate the virus at the appropriated MOI, the TCID_50_ was converted to PFU using a previously reported algorithm^39^. SARS-CoV-2 was handled in a biosafety level 3 facility. NoV GII.4-positive stool was dissolved in phosphate-buffered saline (PBS) at 10% (w/v) by vigorous vortexing. After centrifugation and filtration^38^, the supernatants were prepared for infection.

### qRT-PCR

Total RNA was extracted from the cells using the RNeasy® Mini Kit (Qiagen, Hilden, Germany), and reverse transcription was performed using the SuperScript VILO master mix (Thermo Fisher Scientific). mRNA levels were quantified by fluorescence real-time PCR on a QuantStudio™ 3 System (Thermo Fisher Scientific) using a set of specific primers and the Fast SYBR® Green Master Mix (Thermo Fisher Scientific). *Glyceraldehyde 3-phosphate dehydrogenase* (*GAPDH*) mRNA was used as an internal control to normalize the mRNA levels of each gene. The assays were performed in triplicates. The sets of primers used were as follows:

*GAPDH*, 5′-GTCTCCTCTGACTTCAACAGCG-3′ (forward) and 5′-ACCACCCTGTTGCTGTAGCCAA-3′ (reverse); *LYZ*, 5′-CCGCTACTGGTGTAATGATGG-3′ (forward) and 5′-CATCAGCGATGTTATCTTGCAG-3′ (reverse); *SI*, 5′-TTTTGGCATCCAGATTCGAC-3′ (forward) and 5′-ATCCAGGCAGCCAAGAATC-3′ (reverse); *CHGA*, 5′-AAAGTGTGTCGGAGATGACCTCAA-3′ (forward) and 5′-TCCCTGTGAACAGCCCTATGAATAA-3′ (reverse); *VIL1*, 5′-AGAGCTGGTACCTGTGGATTCC-3′ (forward) and 5′-TGCCCTGCCAAACGTAGAG-3´ (reverse); *LGR5*, 5′-ACTTTGAGGAAGACCTGAAAG-3´ (forward) and 5′-TCCACACTCCAATTCTGATC-3′ (reverse); *MUC2*, 5′-TGTAGGCATCGCTCTTCTCA-3′ (forward) and 5′-GAGTCCATCCTGCTGACCAT-3′ (reverse); *ACE2*, 5′-TCCATTGGTCTTCTGTCACCCG-3′ (forward) and 5′-TACGAGGGTGAACTTGGTCAGC-3′ (reverse); *FURIN*, 5´-GCCACATGACTACTCCGCAGAT-3´ (forward) and 5´-AGACCATCCACCTCCACTTCTC-3´ (reverse); *CTSL*, 5′-GAAAGGCTACGTGACTCCTGTG-3′ (forward) and 5′-CCAGATTCTGCTCACTCAGTGAG-3′ (reverse); *TMPRSS2*, 5′-CCTCTAACTGGTGTGATGGCGT-3′ (forward) and 5′-TGCCAGGACTTCCTCTGAGATG-3′ (reverse); *TMPRSS4*, 5′-GACGAGGAGCACTGTGTCAAGA-3′ (forward) and 5′-GAAACAGGCAGAGAACCAGTTCC-3′ (reverse); *IFNA1*, 5´-AGAAGGCTCCAGCCATCTCTGT-3′ (forward) and 5′-TGCTGGTAGAGTTCGGTGCAGA-3′ (reverse); *IFNA2*, 5′-TGGGCTGTGATCTGCCTCAAAC-3′ (forward) and 5′-CAGCCTTTTGGAACTGGTTGCC-3′ (reverse); *IFNB1*, 5′-CTTGGATTCCTACAAAGAAGCAGC-3′ (forward) and 5´-TCCTCCTTCTGGAACTGCTGCA-3′ (reverse); *IFNG*, 5´-GAGTGTGGAGACCATCAAGGAAG-3′ (forward) and 5′-TGCTTTGCGTTGGACATTCAAGTC-3′ (reverse). Each value is representative of at least three independent experiments and is shown as the mean ± SD from three wells of supernatants of each culture group.

### Immunohistochemical analysis

IEC monolayers were fixed and blocked with a Fixation/Permeabilization Solution Kit (BD Biosciences, NJ, USA) and stained with goat anti-ACE2 (Bio-Techne, MN, USA: AF933, 0.2 mg/mL, 150-fold diluted), rabbit anti-TMPRSS2 (Merck, Darmstadt, Germany: HPA-035787, 40-fold diluted), and mouse anti-ZO-1 (Thermo Fisher Scientific: ZO1-1A12, 1000-fold diluted) antibody. To visualize virus NP and SP, IEC#17 monolayers were infected with SARS-CoV-2 at an MOI of 1.0. After washing the cells at 24 hpi, specimens were fixed and blocked with a Fixation/Permeabilization Solution Kit, and were stained with goat anti-ACE2, mouse anti-TMPRSS2 (Santa Cruz Biotechnology, TX, USA: sc-515727, 0.1 mg/mL, 200-fold diluted), mouse anti-SARS-CoV-2 NP (clone: 3A9, Cell Engineering Corporation), and rabbit anti-SARS-CoV-2 SP (clone: SA39, Cell Engineering Corporation). After washing the primary antibodies, the specimens were stained with appropriate donkey secondary antibodies conjugated with Alexa Fluor 488 or Cy3 or Alexa Fluor 647 (Jackson ImmunoResearch, PA, USA). Samples were fixed with ProLong Diamond Antifade Mountant with DAPI (Thermo Fisher Scientific). Images were captured using a fluorescence microscope (IX83-DSU, OLYMPUS, Tokyo, Japan) and confocal laser microscope (FV3000, OLYMPUS).

### Growth kinetics

Vero cells were seeded in a 24-well plate at 2 × 10^5^ cells per well. The differentiated IEC#17 cells were seeded in a 96-well plate at approximately 1 × 10^5^ cells per well. The virus solution was diluted appropriately with DMEM containing 2% FBS. The cells were washed with PBS and infected with SARS-CoV-2 at an MOI of 0.1 for 1 h at 37 °C. After inoculation, the cells were washed with PBS twice, and 100 µL of DMEM containing 5% FBS or differentiation medium was added for Vero cells or IEC#17, respectively. Supernatants were collected at the indicated time points, and the TCID_50_ was determined.

### Virus titration

The VeroE6/TMPRSS2 cells were seeded in a 96-well plate at 50–70% confluency. The virus was tenfold serially diluted with DMEM containing 2% FBS. The virus at each dilution was inoculated into four wells. The plate was incubated for 3 days at 37 °C until cytopathic effects were visible. The cells were fixed with PBS containing 5% formalin (FUJIFILM Wako Pure Chemical Corporation, Osaka, Japan) and stained with crystal violet (FUJIFILM Wako Pure Chemical Corporation). The TCID_50_ was calculated using the Spearman–Kaerber calculation method. Each value is representative of at least three independent experiments and is shown as the mean ± SD from three wells of supernatants of each culture group. NoV GII.4 propagation was quantified by qRT-PCR using a qPCR Norovirus (GI/GII) Typing Kit (TaKaRa, Shiga, Japan) and the LightCycler 480 System (Roche) in accordance with the manufacturer’s instructions. Each value is representative of at least three independent experiments and is presented as the mean ± SD from 4 to 6 wells of supernatants of each culture group.

### TEM analysis

The differentiated IEC#17 cells were prepared in 2.5% Matrigel-coated Transwell membranes (Corning, NY, USA: 3470) or Cell Desk LF (Sumitomo Bakelite, Tokyo, Japan). The cells were infected with SARS-CoV-2 at an MOI of 0.1. At 24 hpi, the cells were fixed with 2% formaldehyde and 2.5% glutaraldehyde in a 0.1 M sodium phosphate buffer (pH 7.4) and washed three times with the same buffer for 5 min. Cells were post-fixed for 1 h with 1% osmium tetroxide and 1% potassium ferrocyanide in a 0.1 M sodium phosphate buffer (pH 7.4), dehydrated using a graded series of ethanol, and embedded in Epon812 (TAAB, Berks, UK). Ultra-thin Sects. (80 nm) were stained with saturated uranyl acetate and a lead citrate solution. Electron micrographs were obtained with a JEM-1400plus transmission electron microscope (JEOL, Tokyo, Japan).

### Inhibition assay

IEC#17 cells were seeded at a concentration of 1 × 10^5^ per well in a 96-well plate and pretreated with the inhibitors, camostat (Cayman Chemical Company, MI, USA) and nafamostat (Cayman Chemical Company) for 1 h before virus infection. Dimethyl sulfoxide (DMSO; Sigma-Aldrich, MO, USA) was used as a control. After removal of the inhibitors, the cells were washed three times and infected with SARS-CoV-2 at an MOI of 0.1 or 2 × 10^6^ genome equivalents of NoV GII.4 for 1 h at 37 °C. At 48 hpi, the supernatant was collected, and the viral titers of SARS-CoV-2 and NoV GII.4 were measured by the TCID_50_ method and qRT-PCR, respectively. Each value is representative of at least three independent experiments and is presented as the mean ± SD from three wells of supernatants of each culture group.

### Statistical analysis

Two-way ANOVA was used to determine the significant differences in the growth kinetics in IECs. For the other experiments, one-way ANOVA was used. The statistical analysis was conducted using GraphPad Prism 9 software (GraphPad Software).

### Ethics declarations

The experiments using primary human organoids and NoV-positive human stools were performed in accordance with the relevant guidelines and were approved by the human ethical committee of Osaka University (approval #27-5 and #28-3), Nihon University (approval #2022-05), and Wakayama Medical University (approval #3565, #3566, and #3716). We received written informed consent of all participants.

## Supplementary Information

Supplementary Figures.

Supplementary Table 1.

## Data Availability

All relevant data are within the manuscript.
