# Supplementary material for: Effective SARS-CoV-2 replication of monolayers of intestinal epithelial cells differentiated from human induced pluripotent stem cells
Source: Sci Rep. 2023 Jul 18;13:11610. doi: 10.1038/s41598-023-38548-1 (PMC10354114; doi:10.1038/s41598-023-38548-1)
Supplement: Supplementary file 1 — Supplementary Figures. [file 41598_2023_38548_MOESM1_ESM.pdf]

Effective SARS-CoV-2 infection of monolayers of intestinal epithelial cells differentiated from human induced pluripotent stem cells

Shohei Minami,[1] Naomi Matsumoto,[1] Hiroko Omori,[2] Yutaka Nakamura,[3] Shigeyuki Tamiya,[3] Ryotaro Nouda,[1] Jeffery A Nurdin,[1] Moeko Yamasaki,[1] Tomohiro Kotaki,[1] Yuta Kanai,[1] Toru Okamoto,[4, 5] Taro Tachibana, [6, 7] Hiroshi Ushijima,[8] Takeshi Kobayashi,[#1, 5] Shintaro Sato[#1, 3]

[1]Department of Virology, Research Institute for Microbial Diseases, Osaka University, Osaka 565-0871, Japan

[2]Core Instrumentation Facility, Research Institute for Microbial Diseases, Osaka University, Osaka 565-0871, Japan

[3]Department of Microbiology and Immunology, School of Pharmaceutical Sciences, Wakayama Medical University, Wakayama 640-8156, Japan

[4]Institute for Advanced Co-creation Studies, Research Institute for Microbial Diseases Osaka University, Osaka, Japan

[5]Center for Infectious Disease Education and Research, Osaka University, Osaka 565-0871, Japan

[6]Cell Engineering Corporation, Osaka 532-0011, Japan

[7]Department of Bioengineering, Graduate School of Engineering, Osaka Metropolitan University, Osaka 558-8585, Japan

[8]Division of Microbiology, Department of Pathology and Microbiology, Nihon University School of Medicine, Tokyo 173-8610, Japan

#Address correspondence to Takeshi Kobayashi, tkobayashi@biken.osaka-u.ac.jp; Shintaro Sato, shintas@wakayama-med.ac.jp

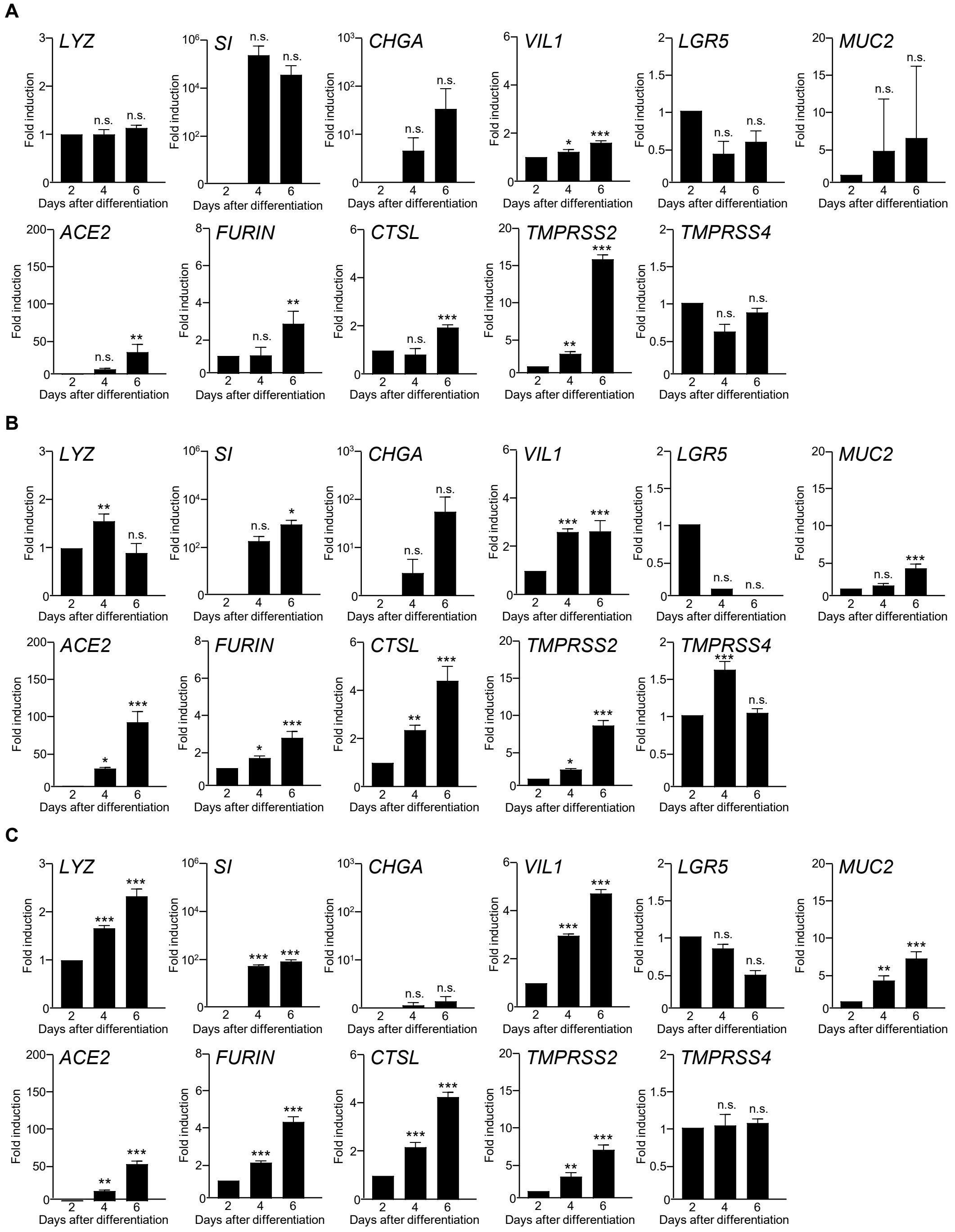

Supplementary Fig. 1 The characteristics of the other IECs. The IEC monolayers were cultured in a differentiation medium from day 2. IECs were collected at the indicated time points. Relative mRNA expression of the indicated genes in IECs during the course of differentiation (days 2, 4, and 6) were determined by qRT-PCR and normalized against the expression of *GAPDH*. Each result was normalized by the expression level at two-day after differentiation. Each value is representative of at least three independent experiments and is shown as the mean ± SD from three wells of cells of each culture group. The significant differences were determined using one-way ANOVA. **(A-C; upper)** The transcription of the major intestinal markers was measured using qRT-PCR. We included *LYZ, SI, CHGA, VIL1, LGR5* and *MUC2* which is the marker of Paneth cell, enterocyte, enteroendocrine cell, IEC, stem cell and goblet cell, respectively in IEC#20 **(A),** IEC#25 **(B)** and IEC#29 **(C)**. **(A-C; lower)** The transcription of the major host factors important for SARS-CoV-2 infection was measured using qRT-PCR. We included ACE2, Furin, CTSL, TMPRSS2 and TMPRSS4 in IEC#20 **(A),** IEC#25 **(B)** and IEC#29 **(C)**. *$0.01<P<0.05$, **$0.005<P<0.01$, ***$P<0.005$

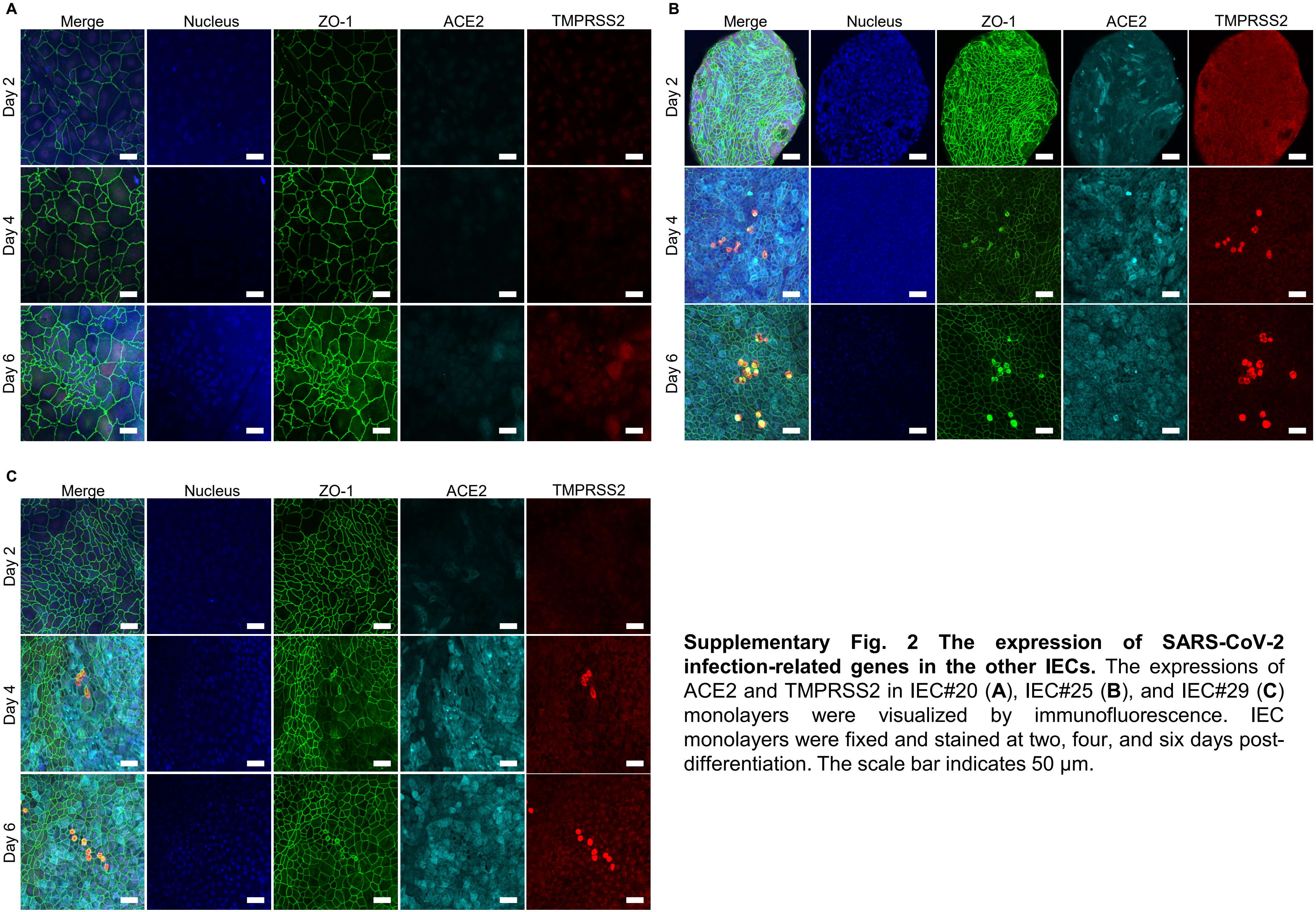

Supplementary Fig. 2 The expression of SARS-CoV-2 infection-related genes in the other IECs. The expressions of ACE2 and TMPRSS2 in IEC#20 (**A**), IEC#25 (**B**), and IEC#29 (**C**) monolayers were visualized by immunofluorescence. IEC monolayers were fixed and stained at two, four, and six days post-differentiation. The scale bar indicates 50 μm.

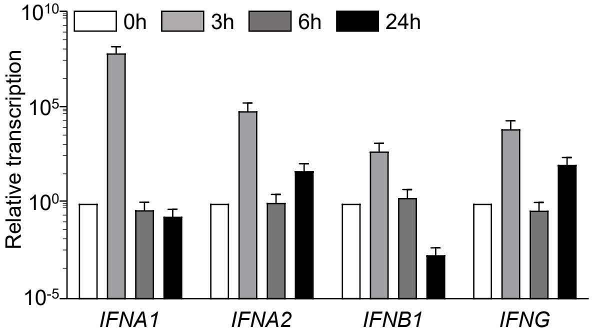

Supplementary Fig. 3 The interferon response of IEC#17 against SARS-CoV-2 infection. IEC#17 were infected with SARS-CoV-2 an MOI of 0.1. The infected cells were collected at the indicated time points. Relative mRNA expression of the indicated genes in IECs were determined by qRT-PCR and normalized against the expression of *GAPDH*. Each result was normalized by the expression level at 0-hour post infection. Each value is representative of at least three independent experiments and is shown as the mean ± SD from three wells of cells of each culture group.
