# Supplementary material for: Effective SARS-CoV-2 replication of monolayers of intestinal epithelial cells differentiated from human induced pluripotent stem cells
Source: Sci Rep. 2023 Jul 18;13:11610. doi: 10.1038/s41598-023-38548-1 (PMC10354114; doi:10.1038/s41598-023-38548-1)
Supplement: Supplementary file 2 — Supplementary Table 1. [file 41598_2023_38548_MOESM2_ESM.docx]

Supplementary Table 1: List of human iPSC used in this study

| Name | Origin | Reprogramming Vector | Gender | Age | IEC No. |
| --- | --- | --- | --- | --- | --- |
| TkDN4-M | neonatal skin | Retrovirus vector | M | neonate | IEC#17 |
| 1231A3 | peripheral blood | Episomal vector | F | 29 | IEC#20 |
| 1383D6 | peripheral blood | Episomal vector | M | 36 | IEC#25 |
| TkPP7 | peripheral blood | Sendai virus vector | M | 40 | IEC#29 |
